# Erinacine A-enriched *Hericium erinaceus* mycelium ameliorates Alzheimer’s disease-related pathologies in APPswe/PS1dE9 transgenic mice

**DOI:** 10.1186/s12929-016-0266-z

**Published:** 2016-06-27

**Authors:** Tzeng Tsai-Teng, Chen Chin-Chu, Lee Li-Ya, Chen Wan-Ping, Lu Chung-Kuang, Shen Chien-Chang, Huang F. Chi-Ying, Chen Chien-Chih, Young-Ji Shiao

**Affiliations:** Institute of Biopharmaceutical Science, National Yang-Ming University, Taipei, 112 Taiwan, Republic of China; Biotechnology Center, Grape King Bio Ltd. Chung-Li, Taoyuan, 320 Taiwan, Republic of China; National Research Institute of Chinese Medicine, Ministry of Health and Welfare, Taipei, 112 Taiwan, Republic of China; Department of Biotechnology and Department of Nursing, HungKuang University, Taichung, 433 Taiwan, Republic of China; Ph.D Program for the Clinical Drug Discovery from Botanical Herbs, College of Pharmacy, Taipei Medical University, Taipei, 110 Taiwan, Republic of China

**Keywords:** Alzheimer’s disease, APPswe/PS1dE9 transgenic mice, Amyloid β, Neurogenesis, Erinacine A-enriched *Hericium erinaceus* mycelia, Insulin-degrading enzyme

## Abstract

**Background:**

The fruiting body of *Hericium erinaceus* has been demonstrated to possess anti-dementia activity in mouse model of Alzheimer’s disease and people with mild cognitive impairment. However, the therapeutic potential of *Hericium erinaceus* mycelia on Alzheimer’s disease remains unclear. In this study, the effects of erinacine A-enriched *Hericium erinaceus* mycelia (HE-My) on the pathological changes in APPswe/PS1dE9 transgenic mouse model of Alzheimer’s disease are studied.

**Results:**

After a 30 day oral administration to 5 month-old female APPswe/PS1dE9 transgenic mice, we found that HE-My and its ethanol extracts (HE-Et) attenuated cerebral Aβ plaque burden. It’s worth noting that the attenuated portion of a plaque is the non-compact structure. The level of insulin-degrading enzyme was elevated by both HE-My and HE-Et in cerebral cortex. On the other hand, the number of plaque-activated microglia and astrocytes in cerebral cortex and hippocampus were diminished, the ratio of nerve growth factor (NGF) to NGF precursor (proNGF) was increased and hippocampal neurogenesis was promoted after these administrations. All the mentioned benefits of these administrations may therefore improve the declined activity of daily living skill in APPswe/PS1dE9 transgenic mice.

**Conclusions:**

These results highlight the therapeutic potential of HE-My and HE-Et on Alzheimer’s disease. Therefore, the effective components of HE-My and HE-Et are worth to be developed to become a therapeutic drug for Alzheimer’s disease.

## Background

Alzheimer’s disease (AD), an age-related progressive neurodegenerative disorder, is characterized by the formation of neurofibrillary tangles, extracellular aggregated amyloid-β (Aβ) plaques, and neural and synaptic loss [1]. The accumulation of Aβ results in neuroinflammation and oxidative stress. Aβ is a normal product of cellular metabolism derived from the amyloid precursor protein (APP) by the successive action of the β- and γ-secretases [2]. The accumulation of Aβ in the brain is then determined by the rate of Aβ generation versus clearance. Clearance can be accomplished via three major pathways: proteolytic degradation, microglia-mediated clearance, receptor-mediated transport across the blood-brain barrier [3].

Compromised neurogenesis presumably takes place earlier than onset of hallmark lesions or neuronal loss, and may play a role in the initiation and progression of neuropathology in AD [4]. Recent studies have shown that brain levels of nerve growth factor (NGF) precursor (pro-NGF) are increased in a stage dependent manner in AD [5], and NGF deprivation leads to cholinergic deficit, Aβ deposition, tau hyperphosphorylation and impaired neurogenesis [6, 7]. Moreover, inflammatory challenge triggered by Aβ induces the production of proinflammatory cytokines by microglia as well as resident astrocytes have profound detrimental effects on adult neurogenesis [8]. Therefore, alteration of ratio of NGF/proNGF, inflammatory challenge and compromising neurogenesis are involved in the developments of AD.

*Hericium erinaceus* is an edible and medicinal mushroom with various pharmacological activities in the prevention of many age-associated neurological dysfunctions, including AD and Parkinson’s disease [9]. Moreover, compounds isolated from its fruiting bodies and mycelia exhibit a potent activity to stimulate NGF expression and secretion in vitro and in vivo [10, 11]. Recent studies demonstrated anti-dementia activity of its fruiting bodies in mice with cognitive deficits induced by Aβ and in people with mild cognition impairment [12, 13]. However, the anti-dementia activity of *Hericium erinaceus* mycelia remained unclear.

In the present study, we investigated the potentials of erinacin A-enriched *Hericium erinaceus* mycelia (HE-My) and it ethanol extracts (HE-Et) on AD-related pathologies in 5 month-old APPswe/PS1dE9 (APP/PS1) double transgenic mouse, a transgenic mouse model of AD expressing two mutations in the human APP as well as two human presenilin 1, exhibiting impaired exploratory behavior, spatial memory and synaptic function [14–16]. Our data suggests that HE-My and HE-Et ameliorated Aβ plaques via increasing the level of insulin degrading enzyme (IDE) and microglia-mediated clearance. Additionally, HE-My and HE-Et also elevated NGF/proNGF ratio. All the alteration may result in promotion of neurogenesis and improving the decline of activity of daily living (ADL) skill.

## Methods

### Sample preparation

**Fig. 1:**
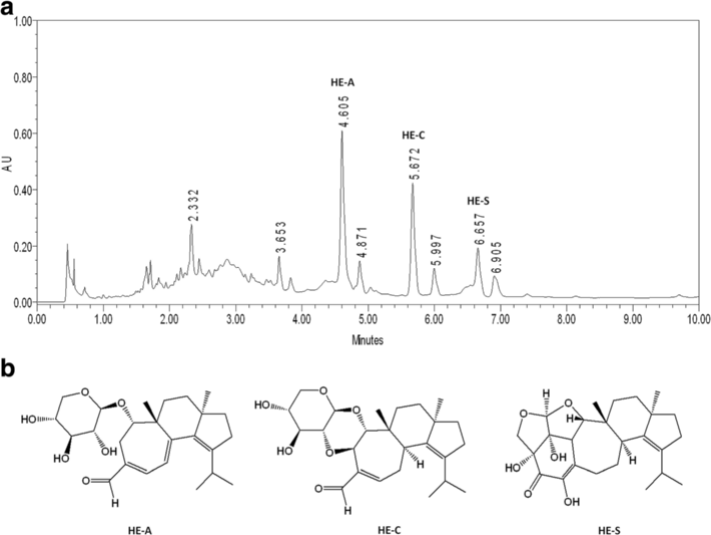
The representative chemical finger prints and the structure of the major components of *Hericium erinaceus* mycelium. **a**. UPLC chromatogram of HE-Et was carried out on a Thermo syncronis C18 (2.1 × 100 mm) column in Waters AcQuity Ultra Performance LC system with a diode array detector, monitored at 210 nm. The mobile phase consisted of 0.1 % phosphate water (**a**) and acetonitrile (**b**) using a gradient elution of 20–55 % B at 0–2 min, 55–90 % B at 2–9 min, 90–100 % acetonitrile at 9–10 min. The flow rate was 0.4 ml/min. Three active components: HE-A, HE-C and HE-S were identified and compared to the standards, and their structure was shown in **b**

*Hericium erinaceus* (BCRC 35669) purchased from Bioresources Collection and Research Center (BCRC) in Food Industry Research and Development Institute (Hsinchu, Taiwan) were maintained and cultivated on potato dextrose agar at 26 °C for 15 days according to the method described earlier [17]. After incubation, a mycelial agar block (1 cm^3^) was removed, transferred to a 2-L Erlenmeyer flask containing 1.3 L synthetic medium (composed of 0.25 % yeast extract, 4.5 % glucose, 0.5 % soybean powder, 0.25 % peptone and 0.05 % MgSO4, adjusted to pH 4.5) and incubated for 5 days at 26 °C on a rotary shaker (120 rpm). The fermentation process was then scaled up from a 2-L shake flask to 500-L and 20-ton fermenters for 5 and 12 days, respectively. At the end of the fermentation process, the mycelia were then harvested, lyophilized, grounded into powder, and stored in a desiccator at room temperature (HE-My). The proximate composition analysis, including ash, total protein, lipids, and carbohydrates of the freeze-dried mycelia were determined according to official AOAC methods. The dry sample was then analyzed by UPLC to evaluate the content in the mycelia based on its dry weight. HE-My was extracted four times with 90 % ethanol under reflux to give the ethanol extract (HE-Et). Their chemical finger prints were determined (Fig. 1). Three active components in HE-Et were identified and were compared to standards. The identification of erinacine A (HE-A), erinacine C (HE-C) and erinacine S (HE-S) in HE-Et using NMR and LC-MS-MS have been previously described [17, 18]. The HE-My (containing 19 mg/g HE-A) and HE-Et (containing 104.4 mg/g HE-A) were dissolved in vehicle (3 % DMSO/10 % cremophor EL (Sigma)/87.5 % D5W (5 % dextrose in water, pH 7.2) with vigorous shaking in order to get a final concentration of 3 mg · ml^−1^ (HE-A) or 30 mg · ml^−1^ (HE-My and HE-Et) before its administration to mice.

### Animal management and administration

The Institutional Animal Care and Use Committee at the National Research Institution of Chinese Medicine approved the animal protocol (IACUC No: 100-A-04 and 102-417-3). The APPswe/PS1ΔE9 double transgenic mouse model (APP/PS1) of AD was purchased from Jackson Laboratory (No. 005864) that expressed a chimeric mouse/human APP695 harboring the Swedish K670M/N671L mutations (APPswe) and human PS1 with the exon-9 deletion mutation (PS1ΔE9). Breeding gender ratio was a male with two females in one cage. Experiments were conducted using wild type (WT) siblings and AD transgenic female C57BL/6 J mice. The animals were housed under controlled room temperature (24 ± 1 °C) and humidity (55–65 %) with 12:12 h (07:00–19:00) light–dark cycle. All mice were provided with commercially available rodent normal chow diet and water *ad libitum*.

To explore the effect of HE-My and HE-Et, 5 months old APP/PS1 mice were fed with HE-My and HE-Et (300 mg/kg/day) for 30 days for pathological test, and 5-bromo-2′-deoxyuridine (BrdU; Sigma) was injected intraperitoneally at 50 mg/kg/day at the last 7 days. For behavior test, 5 months old APP/PS1 mice were fed with HE-My (300 mg/kg/day) for 70–90 days.

### Tissue processing

After administration by oral gavage to 5 month-old mice for 30 days, 5-bromo-2′-deoxyuridine (BrdU; Sigma) was injected intraperitoneally (50 mg/kg/day) at the last 7 days, then the mice were deeply anesthetized with chloral hydrate and then sacrificed by transcardial perfusion with saline at pH 7.4. Mice brain was removed and one hemisphere was homogenized in H-Buffer (320 mM sucrose, 2 mM EDTA, 20 mM Tris-HCl (pH 7.4), 1 mM PMSF, 5 μg/ml leupeptin, 5 μg/ml aprotinin) for Aβ ELISA and immunoblots. Another hemisphere was processed for histochemical and immunohistochemical staining. Dissected brains were immersed in 4 % formaldehyde for 18 h at 4 °C, then cryoprotected in sucrose before being sectioned into 30 μm thick free-floating sections.

### Thioflavin S staining

Thioflavin S (Ths; Sigma) is widely used to detect amyloid plaque deposition [19]. For triple immunofluorescent staining with thioflavin S, Aβ plaque and microglia, dried sections were stained with fresh, filtered 1 % thioflavin S in water for 60 min, and then washed twice with 70 % ethanol for 5 min each, twice with water for 2 min each, twice with PBS for 5 min each. The sections were then incubated in blocking buffer and antibody dilution buffer with corresponding antibodies, as mentioned above.

### Immunohistochemistry

The mice were sacrificed by transcardial perfusion with saline at pH 7.4. One hemisphere of brain was homogenized in H-Buffer (320 mM sucrose, 2 mM EDTA, 20 mM Tris–HCl (pH 7.4), 1 mM PMSF, 5 μg/ml leupeptin, 5 μg/ml aprotinin) for Aβ ELISA and immunoblots. Another hemisphere of brain was processed for immunohistochemical detection. Dissected brains were immersed in 4 % formaldehyde for 18 h at 4 °C, then cryoprotected in sucrose before being sectioned into 30 μm thick free-floating sections. Sections were blocked in blocking buffer (PBS with 3 % normal donkey serum, 1 % bovine serum albumin (BSA) and 0.3 % Triton X-100) for 60 min and incubated overnight at 4 °C in antibody dilution buffer (PBS with 1 % normal donkey serum, 1 % BSA and 0.3 % Triton X-100) with primary antibodies, including mouse monoclonal anti-GFAP antibody (1:300, Millipore) and anti-Aβ1-16 antibody (AB10, 1:300, Millipore), goat anti-Iba-1 antibody (1:300, abcam). Sections were then incubated in antibody dilution buffer containing Hoechst33258 (Invitrogen, 2 μg/ml), Fluorescein isothiocyanate-conjugated donkey anti-mouse IgG and RRX-conjugated donkey anti-rabbit IgG or Alexa Fluor 647-conjugated donkey anti-goat IgG (1:300; Jackson ImmunoResearch) in the dark for 2 h at room temperature. Sections were then washed with PBS containing 0.01 % Triton X-100 and mounted with Aqua Poly/Mount (Polyscience Inc., Warrington, PA, USA). Fluorescent images of immunohistochemistry and ThS staining were taken using a Zeiss LSM 780 confocal microscopy (Jena, Germany). Amyloid plaque deposition was quantified using ImageJ software. The amyloid plaque burden indicates the AB10-reactive or ThS-positive area normalized to the total area.

For detection of BrdU-positive cells, tissue sections were incubated in 10 mM sodium citrate (pH 6.0) at 80 °C for 30 min, and subsequently in 2 M HCl at 37 °C for 30 min. The sections were then incubated in blocking buffer and antibody dilution buffer with primary antibodies, including mouse monoclonal antibodies to BrdU (1:500, Senta Cruz) and rabbit polyclonal antibody to doublecortin (DCX, 1:500, abcam), and corresponding secondary antibodies, as mentioned above. The number of BrdU- or DCX-positive in the subgranular zone (SGZ) of dentate gyrus was quantified and revealed as the linear density of BrdU- or DCX-positive cells per millimeter of SGZ.

### Quantification of ThS- and AB10-stained plaques

The quantification of ThS- and AB10-stained plaques was conducted individually. At least 3 coronal brain sections from each mouse were used for analysis. Each image was adjusted to the threshold for pixel detection (threshold setting for ThS-positive signal is 60; threshold setting for AB10-positive signal is 40). To eliminate background, particle less than 100 pixels (approximately 70 μm^2^) was excluded. Occupied area by ThS- or AB10-stained signal was divided by the full area of interest, including cortex and hippocampus, and represented as percentage.

### Mesurement of Aβ_1–42_ levels

Two-step sequential extraction of the brain Aβ using 2 % SDS and 70 % formic acid (FA; Sigma) was processed as described previously [20]. Briefly, cortical homogenate was mixed with equal volume of 4 % SDS in H-buffer containing protease inhibitor. The sample was then sonicated and centrifuged at 100,000 × *g* for 60 min at 4 °C. The supernatant was considered SDS-soluble fraction. The SDS-insoluble pellet was further resuspended in 70 % FA and centrifuged at 100,000 × *g* for 60 min at 4 °C. The supernatant was collected and neutralized with 1 M Tris, pH 11. SDS-soluble and SDS-insoluble fractions were stored at −80 °C until sandwich ELISA analysis. Aβ level was measured by a sensitive fluorescence based sandwich ELISA assay using a kit (Human β- Amyloid 1–42, Invitrogen KHB3442). The detailed experiments were performed according to the manufacturer’s protocol.

### Immunoblots

For Western blot analysis, samples (30 μg protein) were separated by sodium dodecyl sulfate-polyacrylamide gel electrophoresis and were then transferred to PVDF membranes. The primary antibodies used were as follows: rabbit anti-Iba-1 antibody (Wako), rabbit anti-NGF antibody, rabbit anti-GFAP antibody, mouse anti-APP antibody, rabbit anti-CTF antibody, rabbit anti-NEP antibody, rabbit anti-IDE antibody (Millipore) and mouse anti-β-actin antibody (Novus Biologicals). The secondary antibodies were anti-rabbit IgG antibody conjugated with horseradish peroxidase (HRP; GE Healthcare) and anti-mouse IgG antibody conjugated with HRP (Jackson ImmunoResearch). Enhanced chemiluminescence detection reagents (GE Healthcare) were used for detection. Bands were quantified using Fujifilm LAS-3000 Luminescent Image Analyzer (Tokyo, Japan).

### Nesting test

After oral gavage administration for 81 days, mice were assessed for nesting task as described previously [21]. Two nestlets (approximately 5 g) were placed into cage at 1 h before dark cycle, and then the nest score and the weight of unshredded nestlets were determined after overnight. The nest quality was assessed on a 1–5 rating scale, with 1, nestlet was not noticeable touched; 2, nestlet was partially shredded and scattered on the floor; 3, nestlet was mostly shredded but only flat nest was build; 4, nest was identifiable but flat; and 5, nest was build in a burrow.

### Statistical analysis

The results are expressed as the mean ± standard deviation (S.D.) and were analyzed by analysis of variance (ANOVA) with post-hoc Bonferroni multiple comparisons tests.

## Results

### HE-My and HE-Et reduce the number of AB10-stained plaque in cerebral cortex and hippocampus of APP/PS1 mice

In APP/PS1 transgenic mice model, plaques are well established and visible at 6 months old. Therefore, 5 months-old APP/PS1 mice were fed with 300 mg/kg/day HE-My or HE-Et for 30 days to explore their effects on plaque burden by double staining with AB10-antibody and ThS to observe the structure of whole plaque and its compact core, respectively. The number of AB10-stained plaque with diameter bigger than 100 pixel (68 μm) in cerebral cortex and hippocampus of APP/PS1 mice were counted.

**Fig. 2:**
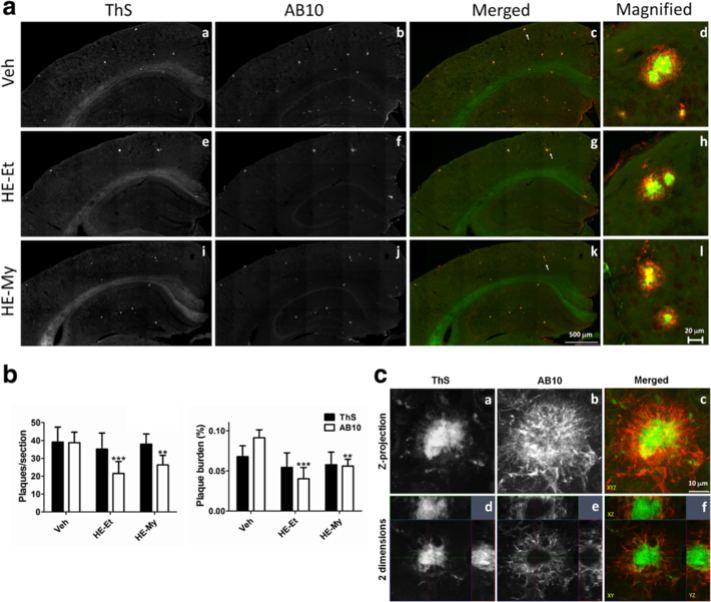
HE-Et and HE-My reduce amyloid plaque burden in the area include the cerebral cortex and hippocampus of APP/PS1 mice. Five month-old APP/PS1 mice were orally administered with vehicle (Veh, *n* = 6), HE-Et (*n* = 5) and HE-My (*n* = 6) for 30 days. **A**. The representative fluorescent images of amyloid plaques detected by thioflavin S (ThS) staining (*white* in a, e and i, and *green* in c, d, g, h, k and l) and immunohistochemical staining with AB-10 antibody (white in b, f and j, red in c, d, g, h, k and l) in the area including parietal cortex and hippocampus. Sale bar: 500 μm. A typical plaque is magnified and shows in the right side of each image. Sale bar: 20 μm. **B**. shows both the plaque number and burden in ThS- and AB-10-stained semi-cerebral sphere calculated by image analysis software. Plaque burden is displayed as a percentage of the area occupied by ThS- or AB-10-stained signal in the full area of interest. **C**. The structure of amyloid plaque in the cerebral cortex of APP/PS1 mice. The amyloid plaque in cerebral cortex of 6 months-old APP/PS1 mice was detected by ThS-staining (*white* in a and d, and *green* in c and f) and immunohistochemical staining with AB-10 antibody (*white* in b and e, and *red* in c and f). *Upper panels* show the representative Z-projection (3 dimensions, XYZ, A-C) of a typical plaque. *Lower panels* show the 2 dimensional images (XY, XZ, and YZ, D-F). The image indicated by *arrow* indicated the putative plaque unit. Sale bar: 10 μm. Single fluorescent images were presented as grayscale to enhance resolution

The treatment did not significantly change the body weight, suggesting that these treatments have no obvious side effect (data not shown). However, the number and burden of whole plaque stained by AB-10, but not compact core stained by ThS, were significantly reduced after 30-day administration of HE-My or HE-Et (Fig. 2a and b). Compared with vehicle-treated mice, HE-My treatment decreased burden and number of whole plaque by 38.6 ± 9.3 % and 34.8 ± 13.4 %, respectively. More effectively, HE-Et treatment decreased burden and number of whole plaque by 55.8 ± 15.2 % and 43.5 ± 15.6 %, respectively.

To identify the detail structure of whole plaque and its compact core, the image of the double stained plaques were displayed in both maximum projection of 3-dimensional stack (Fig. 2C, a–c) and confocal cross sections in xy, yz, and xz (Fig. 2C, d–f). The result showed that a typical plaque was composed by a ThS-stained compact core and an AB10-imunostained filamentary structure, which surround the compact core.

### HE-My and HE-Et reduce the size of AB10-stained plaque in cerebral cortex and hippocampus of APP/PS1 mice

**Fig 3:**
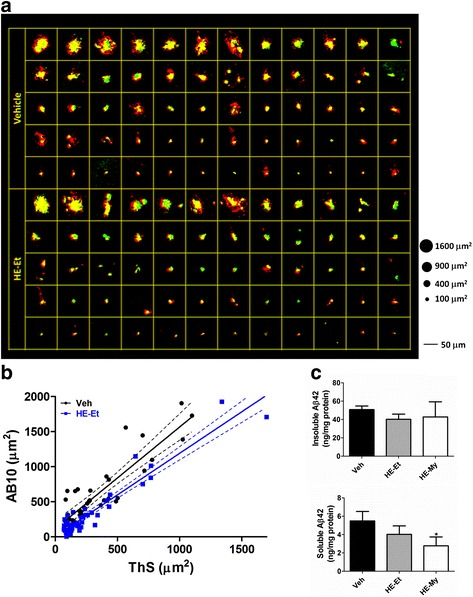
HE-Et reduces size of AB-10-stained plaque in the area including cerebral cortex and hippocampus of APP/PS1 mice. **a**. The representative images of ThS (*green*)- and AB-10 (*red*)-stained plaques (60 for each) selected in order of decreasing size from the area include the cerebral cortex and hippocampus of vehicle- and HE-Et-treated APP/PS1 mice. **b**. A scatter plot of ThS- and AB-10-stained areas from representative samples with vehicle and HE-Et treatments (*solid lines*: linear regression lines; *dashed lines*: 95 % confidence intervals; *R* ^2^ = 0.8224 for vehicle and *R* ^2^ = 0.9079 for HE-Et). **c**. The levels of soluble and insoluble Aβ1-42 determined by ELISA. The results are the mean ± S.D. Significant differences between treated and control (Veh) groups are indicated by *, *p* < 0.05

Sixty double stained plaques of the representative slice of vehicle- and HE-Et-treated mice were selected by size to reveal the difference of plaque conformation (Fig. 3a). The result showed that the non-compact structure of plaques was significantly reduced by the treatment of HE-Et. A scatter plot of the plaque size in μm^2^ with compact core (ThS-staining) against whole plaque (AB-10-staining) was plotted to reveal the morphological alteration of an individual plaque before and after HE-Et treatment. The result showed that the HE-Et modulated structure confined in the AB10-stained non-compact part of whole plaque but not the ThS-stained compact core (Fig. 3b). The similar result was found in the HE-My treated mice (data not shown).

For examining the involvement of Aβ_1–42_ in the plaque structure, soluble and insoluble Aβ were isolated from the cortex of APP/PS1 mice and the Aβ level was detected using Aβ_1–42_-ELISA analysis (Fig. 3c). The results showed that administration of HE-My significantly reduced the level of soluble, but not insoluble Aβ_1–42_. Alternatively, no significant effect of HE-Et on the level of both soluble and insoluble Aβ was observed.

### HE-Et and HE-My reduce Aβ-associated glial cells in APP/PS1 mice

**Fig. 4:**
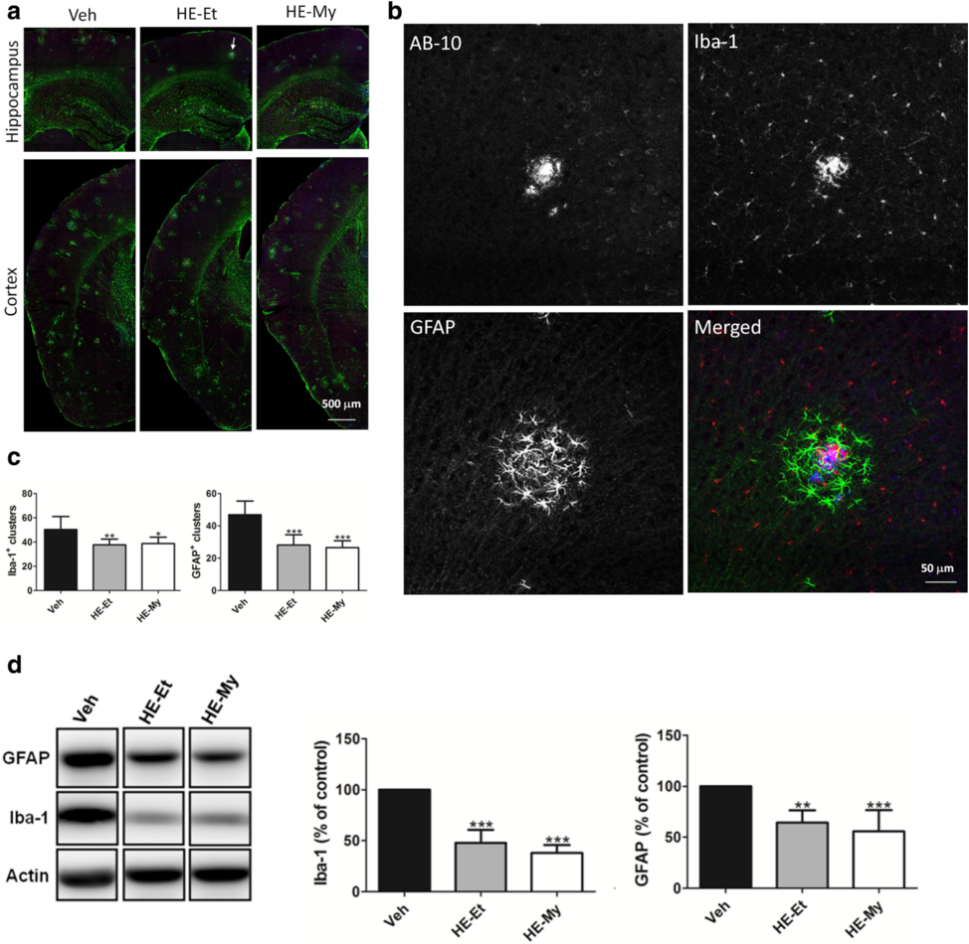
HE-Et and HE-My alleviate amyloid plaque-associated glial activation in the area including hippocampal and cortex of APP/PS1 mice. Five month-old APP/PS1 mice were orally administered with vehicle (Veh, *n* = 6), HE-Et (*n* = 5) and HE-My (*n* = 6) for 30 days. Amyloid plaques were detected by immunohistochemical staining with AB-10 antibody (*blue*). Microglia and reactive astrocytes were detected by immunohistochemical staining with Iba-1 antibody (*red*) and GFAP antibody (*green*), respectively. **a**. The representative immunostaining images of hippocampus and partial cortex. Sale bar: 500 μm. **b**. The enlarged views of a typical cluster which is indicated in panel **a** (*arrow*). Sale bar: 50 μm. **c**. Both the number of microglial and astroglial cluster in semi-cerebral sphere calculated by image analysis software. **d**. The immunoblotting of Iba-1 and GFAP. Representative immunoblots of the Iba-1, GFAP and β-actin were showed (*left panel*). The relative levels of Iba-1 and GFAP were expressed as percentage of control (Veh) (*right panel*). The results are the mean ± S.D. Significant differences between treated and control (Veh) groups are indicated by *, *p* < 0.05; **, *p* < 0.01; ***, *p* < 0.001

Next, we evaluated the effects of HE-Et and HE-My on plaque-associated clusters of activated microglia and reactive astrocytes by immunohistochemical staining with AB-10, Iba-1 and GFAP antibodies, respectively. The image revealed that both the activated astrocyte and microglia formed cluster surrounding plaque in the area including cortex and hippocampus (Fig. 4a and b). HE-Et treatment decreases the number of Iba-1-positive microglia cluster and GFAP-positive astrocyte cluster by 25.2 ± 9.4 % and 40.0 ± 13.5 %, respectively (Fig. 4c). HE-My treatment decreases the number of microglia and astrocyte cluster by 19.4 ± 13.9 % and 43.3 ± 9.0 %, respectively (Fig. 4c). To determine the total reducing of microglia activation and astrocytes reactivity by HE-My and HE-Et, the cortex homogenates of the treated mice were examined by immunoblot using anti-Iba-1 and anti-GFAP antibodies. The result showed that the level of microglia activation was decreased by HE-Et and HE-My to 47.9 ± 12.9 % and 37.9 ± 7.9 % of control, respectively (Fig. 4d). In contrast, the level of astrocytes reactivity was reduced to 64.3 ± 12.1 % and 55.7 ± 20.8 % of control.

### HE-Et and HE-My promote the level of Aβ degrading protein in cortex of APP/PS1 mice

**Fig 5:**
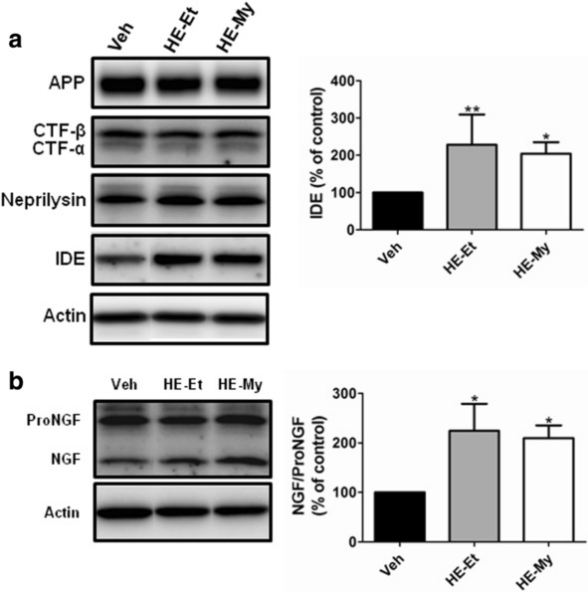
Effects of HE-Et and HE-My on the level of the proteins regulating Aβ accumulation and the level of NGF in cerebral cortex of APP/PS1 mice. Five months-old APP/PS1 mice were orally administered with vehicle (Veh, *n* = 6), HE-Et (*n* = 5) and HE-My (*n* = 5) for 30 days. Cortex was removed and homogenized, and the proteins involving in amyloidogenesis and amyloid clearance and NGF maturation in lysates were analyzed by immunoblotting. **a** The representative immunoblots of APP, CTF-α, −β, IDE, neprilysin and β-actin (*left panel*). The level of APP, CTF-α and -β, IDE and neprilysin was presented as percentage of control (Veh) (*right panel*). **b** The representative immunoblots of the proNGF, NGF and β-actin (*left panel*). The relative level of NGF/proNGF ratio was presented as percentage of control (Veh) (*right panel*). The results are the mean ± S.D. Significant differences between treated and control (Veh) groups are indicated by *, *p* < 0.05; **, *p* < 0.01

To understand the mechanisms by which HE-Et and HE-My reducing Aβ plaque in APP/PS1 mice, we assessed the proteins involving APP processing and Aβ degradation. APP processing was examined by the levels of full length APP, and C-terminal fragments (CTF)-α and CTF-β. The result revealed that HE-Et and HE-My treatment did not affect the levels of APP, CTF-α and CTF-β. Next, we detected the levels of Aβ degrading enzymes including IDE and neprilysin. The result showed that HE-Et and HE-My treatment increased the level of IDE in cortex by 127.7 ± 81.4 % and 103.9 ± 30.9 %, respectively (Fig. 5a). However, no effect on the level of neprilysin was observed.

### HE-Et and HE-My increase NGF/proNGF ratio and promote hippocampal neurogenesis in APP/PS1 mice

The levels of NGF and proNGF were analyzed by immunoblotting. HE-Et and HE-My significantly increase NGF/proNGF ratio by 124.7 ± 54.1 % and 109.9 ± 25.7 %, respectively (Fig. 5b).

**Fig. 6:**
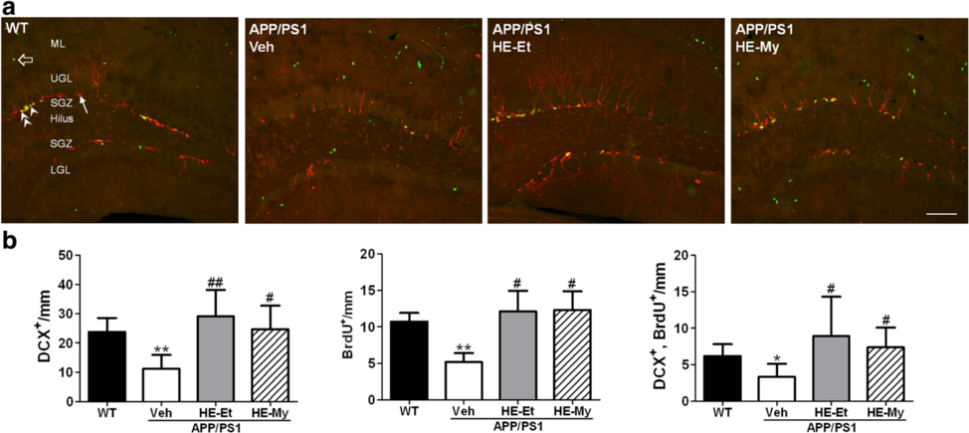
HE-My and HE-Et promotes hippocampal neurogenesis in APP/PS1 mice. Five months old wild type (WT) or APP/PS1 mice were orally administered with vehicle (Veh, *n* = 6), HE-Et (*n* = 6) and HE-My (*n* = 6) for 30 days. Hippocampal neurogenesis was detected by immunohistochemical staining with doublecortin (DCX) antibody (*red*) and BrdU antibody (*green*). **a**. The representative immunostaining images of dentate gyrus in wild type (WT, *n* =7) mice and APP/PS1 mice treated with vehicle (Veh), HE-Et and HE-My. *Arrow* indicates DCX-labeled newly born neuron; *arrow head* indicates proliferating type 2 neuroprogenitor; *double arrow head* indicates the newly born neuron immediately after proliferation; *hollow arrow* indicates proliferating cells other than neuroprogenitor. Scale bar: 100 μm. ML, molecular layer; UGL, upper blade granular cell layer; LGL, SGZ, subgranular zone; lower blade granular cell layer. **b**. The number of BrdU positive cells, doublecortin positive cells and the cells with double labeling (cell number/mm SGZ). The results are the mean ± SD. Significant differences between wild type (WT) and APP/PS1 groups are indicated by *, *p* < 0.05. Significant differences between treated and control (Veh) groups are indicated by #, *p* < 0.05; ##, *p* < 0.01

We hypothesized that the increased NGF/proNGF ratio and declined microglial activation may promote hippocampal neurogenesis. Therefore, newly born granular neurons and proliferating type 2 progenitors in the subgranular zone (SGZ) was assessed by immunohistochemical staining using anti-DCX and anti-BrdU antibody, respectively. We found that the number of both newly born granular neurons (DCX-positive) and proliferating type 2 progenitors (BrdU-positive) were declined in APP/PS1 mice as compared with the wild type mice (Fig. 6). However, the declined number of the DCX-, BrdU-, and BrdU/DCX-double positive neurons were recovered after the treatment of HE-My and HE-Et.

### HE-My recovers activity of daily living decline in APP/PS1 mice

**Fig. 7:**
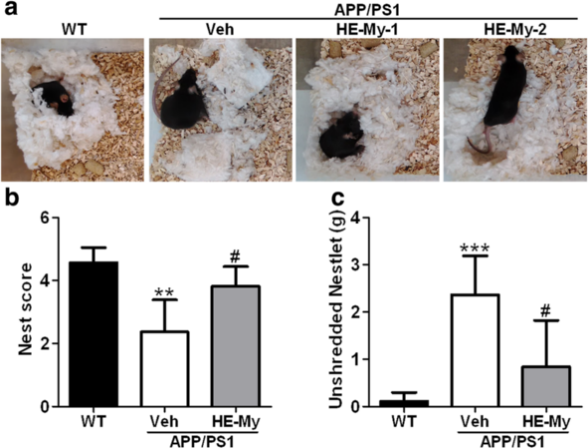
The deficit on nest construction displayed by APP/PS1 mice were improved by HE-My treatment. Five months old wild type (WT) or APP/PS1 mice were orally administered with vehicle (Veh, *n* = 6) or HE-My (*n* = 6) for 81 days and then nest construction test were performed. The representative images of the nest construction (**a**), the nest score (**b**) and unshredded nestlet (**c**) were shown. The results are the mean ± SD. Significant differences between WT (*n* = 8) and APP/PS1 groups are indicated by **, *p* < 0.01; ***, *p* < 0.001. Significant differences between treated and control (Veh) groups are indicated by #, *p* < 0.05

Nesting behaviors engage a broad network of brain regions; have previously been applied on evaluating the daily living skills of AD transgenic mice. Therefore, nesting task was performed. The result showed that APP/PS1 mice had lower nesting score and less nestlet shredding than WT mice. However, HE-My-treated APP/PS1 mice recovered the decline of both nesting score (Fig. 7a and b) and nestlet shredding (Fig. 7a and c).

## Discussion

Our results reveal that the administrations of HE-Et or HE-My to APP/PS1 mice for 30 days eliminated amyloid plaque burden, prevented recruitments and activations of plaque-associated microglia and astrocytes, promoted the expression of IDE, the NGF/proNGF ratio, and the proliferation of neuron progenitors and the number of newly born neurons in dentate gyrus. Finally, we found that the administrations of HE-My for 81 days recovered the deficits on activity of daily living skills in APP/PS1 mice.

Our previous study shows that 300 mg/kg HE-My display the best/highest neuroprotective activity against ischemia reperfusion injury [22]. The safe dose of erinacine A-enriched *Hericium erinaceus* was as high as 3000 mg/kg in a 28-day oral feeding study in Sprague-Dawley rats [17]. Furthermore our previous report showed that 30 mg/kg erinacine A and S process similar activity to reduce Aβ deposition and increase IDE expression [18]. HE-My and HE-Et contain 19 mg/g and 104.4 mg/g of HE-A, respectively. Hence, it is likely that the ingredients form HE-My or HE-Et process similar activity and HE-My or HE-Et (300 mg/kg/day) reach ceiling effect on Aβ clearance. Therefore, we chose 300 mg/kg to test its effect.

Previous reports show that female APP/PS1 mice have more deposition of amyloid plaque than that of male APP/PS1 mice [23–25], and both male and female APP/PS1 mice reveal the similar impairment in spatial learning and memory [23]. Additionally, both genders reveal good activity to build nest [26, 27]. Therefore, we think female APP/PS1 mice is also suitable as a model to evaluate the effect of treatment on AD-like pathology.

Previous studies showed that *H. erinaceus* mycelia and its components possess the activity to stimulate NGF expression and secretion in vitro and in vivo [10]. In our present study, it is evidenced that HE-My and its components increase the NGF/proNGF ratio (Fig. 5b). Recent studies pointed out that the NGF metabolic dysfunction is associated with AD and Down syndrome [28]. Mature NGF is derived from proNGF by some convertases, such as plasmin and furin, and degraded by matrix metalloprotease 9. However, it is still unclear whether *H. erinaceus* mycelia and its components induce the expression of plasmin and furin, or reduce the expression of MMP-9.

The structure of amyloid plaque [29] and the sequential clustering of plaque-associated activated microglia and reactive astrocytes [30] have been studied by some previous works. Those works suggested that the accumulation of Aβ may lead to abnormal neuroinflammation and oxidative stress, and the microglial activation is highly associated with the accumulation of Aβ. In our present study, it is evident that the glial cluster is constructed by at least four sequential layers (i.e. amyloid compact core with radiating arm; amyloid none β-pleated sheet filamentary structure; microglial clustering; and astroglial clustering) (Figs. 2c and 4b). The activity of HE-My and HE-Et to reduce the filamentary structure of amyloid plaque burden in both cortex and hippocampus is a novel finding.

Although Aβ staining by both ThS and AB10 cannot functionally distinguish between increased phagocytosis and impaired degradation, our results showed that HE-My and HE-Et substantially altered the characteristics of Aβ plaque deposition in the APP/PS1 mice (Figs. 2 and 3). HE-My and He-Et significantly reduced the non-compact structure of amyloid plaque (Fig. 3b). Additionally, HE-My-treated APP/PS1 mice showed a 40 % reduction in brain concentrations of soluble forms of Aβ_1–42_ (Fig. 3c).

AD is characterized by elevated levels of Aβ that are produced by β- and γ-secretases [31]. As a corollary, any approach aimed at blocking the production of Aβ by interfering with these enzymes could be seen as a valid therapeutic strategy. On the other hand, activation of α-secretase is another strategy to block the production of Aβ. In our present study, HE-My and HE-Et attenuated the AB10-stained non-compact plaque structure and reduced the level of soluble form Aβ_1–42_ may be attributable to the inhibition of β- or γ-secretase or activation of α-secretase. For determining this effect, the levels of APP, CTF-α and -β were measured. However, the outcome was negative. However, HE-Et and HE-My were found to promote IDE expression suggesting that Aβ clearance by degradation might be involved in the Aβ reduction effect. IDE is the degrading enzyme for Aβ and APP intracellular domain (AICD) [32, 33]. IDE is located in the cytosol and mitochondria [34], and a small proportion of IDE also secreted out from cells [35]. Agonists of the PPARγ pathway have been shown to enhance Aβ clearance by a mechanism that appeared to implicate IDE. Moreover, Notch signaling pathway may also regulate IDE expression [36]. The data establishing an involvement of IDE in Aβ degradation in vivo are very strong. Hence, up-regulation of IDE activity remains to be a therapeutic choice [37]. IDE knockout mice and rat with partial loss-of-function mutations in IDE reveal cerebral Aβ accumulation by 50 % [38]. In contrast, previous work demonstrated that two-fold increase of IDE via transgenic over-expression reduces obviously 50 % Aβ deposition in APP transgenic mice [37]. Therefore, it is likely that the two-fold increase of IDE expression after HE-My or HE-Et treatment may attenuate amyloid plaque burden. It has been reported that with age or after some insults, level of IDE in the brain reduce significantly [39]. In our present study, APP/PS1 transgenic mice, IDE was up-regulated as compared to the wild type mice (Data not shown). This result is consistent with the previous report that in APPswe/PSEN1(A246E) transgenic mice, IDE was up-regulated by Aβ [40].

Recent evidence suggests that impaired clearance may be the driving force behind sporadic AD [41]. In addition to phagocytosis, microglia also contribute to Aβ clearance through proteolytic enzymes, including IDE and neprilysin [42]. In the present study, we found that HE-My and HE-Et promote IDE expression, suggesting that Aβ clearance by IDE degradation may be involved in the Aβ reduction effect. PPARγ pathway and Notch signaling have been found to enhance IDE expression in neurons [43, 44]. However, more study is required to reveal the mechanism mediates HE-My and HE-Et promoted IDE expression.

HE-My and HE-Et were found to promote hippocampal neurogenesis (Fig. 6). Recent study found that APP could function to influence neurogenesis via its two separate domains, sAPPα and AICD. The sAPPα was shown to be neuroprotective and important to neurogenesis, whereas AICD was found to negatively modulate neurogenesis [45]. In our present study, however, administration of HE-Et and HE-My did not significantly decreased the level of CTF-α and CTF-β, which are substrates for γ-secretase to produce AICD. Therefore, neurogenesis may not be promoted by the decrease of AICD after the administration of HE-Et or HE-My. On the other hand, inflammatory challenge triggered by Aβ induces the production of proinflammatory cytokines by microglia as well as resident astrocytes have profound detrimental effects on adult neurogenesis [46].

We focus on species-specific nest building activity because those are spontaneous hippocampus-dependent behaviors [21] that have been proposed to be equivalent to activities of daily living (ADL) skills in humans [47], and the loss of ADL skills is the warning sign of AD in clinical [48]. Moreover, the deficits in nesting behaviors have been shown in APP/PS1 mice [26, 49] and Tg2576 transgenic mice [50]. In our unpublished data, HE-Et or erinacine A also improved the nesting behavior with similar activity. Besides in hippocampus, nesting tests are sensitive to lesions in medial prefrontal cortex and various regions including septum, respectively [21, 26]. We found that HE-My restored nesting behavior (Fig. 7), suggesting that HE-My may improve the impairment in multiple brain regions in APP/PS1 mice.

## Conclusion

Our results suggested that HE-My reduced cortical and hippocampal amyloid plaque burden through increasing the level of IDE, and increase NGF maturation and hippocampal neurogenesis in APP/PS1 mice. We also show that HE-My recovers behavioral deficits in APP/PS1 mice. These findings raise the possibility that HE-My may have therapeutic potential for treating AD as well as the other neurodegenerative diseases.

## Abbreviations

AD, Alzheimer’s disease; ADL, activities of daily living; AICD, APP intracellular domain; APP, amyloid precursor protein; APP/PS1, APPswe/PS1ΔE9; Aβ, β-amyloid peptide; BDNF, brain derived neurotrophic factor; BrdU, 5-bromo-2′-deoxyuridine; CTF, C-terminal fragment; DCX, doublecortin; FA, formic acid; HE-Et, ethanol extracts of HE-My; HE-My, *H. erinaceus* mycelia; IDE, insulin-degrading enzyme; MCI, mild cognitive impairment; NGF, nerve growth factor; proNGF, NGF precursor; PS1, presenilin 1; SGZ, subgranular zone; ThS, thioflavin S; WT, wild type
